# Municipalisation Proposal

**Published:** 1920-03-20

**Authors:** 


### Municipalisation Proposal.

The centralisation of distribution is a problem which the Manchester City Council will have under discussion next month, when it will debate a proposal to municipalise the city's supply. Probably this plan would find greater favour than Dr. Lauder's alternative of centralisation in the hands of two or three great firms. The public is very suspicious of anything savouring of trusts, after the revelations of the past few months. The Government's intention is to invest local authorities with powers such as those which Manchester is to consider, and whatever the case may be against municipalisation, there seems no doubt that a municipality would be able to exercise far greater power—as a big purchaser—than is exercised at present over all stages of production, transit, and distribution. The cost of buying out the milk dealers in Manchester is estimated at about £500,000, and doubtless opposition will be centred on the cost of purchase, the question of the efficiency of local bureaucracies, and the whole political question of dispensing with individual enterprise. There is this to be said, however, about the municipalisation of a vital food, that there is much sharper local power of opinion to stimulate efficiency—if it wants to stimulate it—and where it is a food that enters every home, interest in its control is not likely to be dormant. Ratepayers and consumers, in any event, have far greater power over a town or city council than over a multiplicity of dealers. The alternative is strict supervision by inspection, but this would probably be more costly. In fact the Health Committee of the Manchester Corporation is of opinion that municipalisation would effect a saving of £100,000 a year in the cost of distribution, a figure which would far exceed the loan charges and permit reduction of price for the consumers.

